# Insight into Metabolic ^1^H-MRS Changes in Natalizumab Induced Progressive Multifocal Leukoencephalopathy Brain Lesions

**DOI:** 10.3389/fneur.2017.00454

**Published:** 2017-09-05

**Authors:** Ruth Schneider, Barbara Bellenberg, Robert Hoepner, Gisa Ellrichmann, Ralf Gold, Carsten Lukas

**Affiliations:** ^1^Department of Neurology, St. Josef Hospital, Ruhr University Bochum, Bochum, Germany; ^2^Department of Diagnostic and Interventional Radiology and Nuclear Medicine, St. Josef Hospital, Ruhr University Bochum, Bochum, Germany

**Keywords:** ^1^H magnetic resonance spectroscopy, multiple sclerosis, natalizumab, progressive multifocal leukoencephalopathy, PML lesions, immune reconstitution inflammatory syndrome

## Abstract

**Background:**

Progressive multifocal leukoencephalopathy (PML) is a severe complication of immunosuppressive therapies, especially of natalizumab in relapsing–remitting multiple sclerosis (MS). Metabolic changes within PML lesions have not yet been described in natalizumab-associated PML in MS patients.

**Objective:**

To study metabolic profiles in natalizumab-associated PML lesions of MS patients by ^1^H magnetic resonance spectroscopy (^1^H-MRS) at different stages during the PML course. To assess changes associated with the occurrence of the immune reconstitution inflammatory syndrome (IRIS).

**Methods:**

20 patients received ^1^H-MRS and imaging at 3 T either in the pre-IRIS, IRIS, early-post-PML, or late post-PML setting. Five of these patients received individual follow-up examinations, including the pre-IRIS or IRIS phase. Clinical worsening was described by changes in the Karnofsky Performance Scale (KPS) and the expanded disability status scale (EDSS) 1 year before PML and scoring at the time of ^1^H-MRS.

**Results:**

In PML lesions, increased levels of the Lip/Cr ratio, driven by rising of lipid and reduction of Creatine, were found before the occurrence of IRIS (*p* = 0.014) with a maximum in the PML–IRIS group (*p* = 0.004). By contrast, marked rises of Cho/Cr in PML lesions were detected exclusively during the IRIS phase (*p* = 0.003). The Lip/Cr ratio decreased to above-normal levels in early-post-PML (*p* = 0.007, compared to normal appearing white matter (NAWM)) and to normal levels in the late-post-PML group. NAA/Cho was reduced compared to NAWM in the pre-IRIS, IRIS, and early-post-PML group. In NAA/Cr, the same effect was seen in the pre-IRIS and early-post-PML group. These cross-sectional results were confirmed by the individual follow-up examinations of four patients. NAA/Cho, Cho/Cr, and the lipid rise relative to NAWM in PML lesions were significantly correlated with the residual clinical worsening (KPS change) in post-PML patients (Spearman correlations ρ = 0.481, *p* = 0.018; ρ = −0.505, *p* = 0.014; and ρ = −0.488, *p* = 0.020).

**Conclusion:**

^1^H-MRS detected clinically significant dynamic changes of metabolic patterns in PML lesions during the course of natalizumab-associated PML in MS patients. Lip/Cr and Cho/Cr may provide additional information for detecting the onset of the IRIS phase in the course of the PML disease.

## Introduction

Progressive multifocal leukoencephalopathy (PML) occurs in immunocompromised patients as an opportunistic infection of the central nervous system caused by the John Cunningham Virus (JCV). Patients with a human immunodeficiency virus (HIV) infection are mainly affected (1), but it also occurs as a rare side effect in natalizumab-treated patients with relapsing–remitting multiple sclerosis (RR-MS) (2). The reactivation of the JCV causes a lytic infection of astrocytes, oligodendrocytes, and neurons resulting in severe demyelination (3). Magnetic resonance imaging (MRI) techniques are essential for the detection of early asymptomatic stages of PML (4) and the immune reconstitution inflammatory syndrome (IRIS) (5). Early recognition of PML by MRI plays a crucial role for treatment decision-making and clinical outcome (4, 6). Even though there are limited data for the prognostic value of MRI regarding the clinical outcome of PML patients, higher survival rates could be demonstrated in more localized manifestations of the disease (unilobar and multilobar brain involvement) in contrast to widespread PML attack within the brain’s white matter (7, 8). Longitudinal observations have shown that functional disability in PML survivors usually stabilizes at reduced levels after about 6 months and tends to stay stable beyond 18 months after PML diagnosis unless new MS relapses occur (7, 9).

In HIV-related PML, MRI-based monitoring of PML lesions in the course of the disease plays an important role with regard to detection of IRIS (10). The occurrence of IRIS in natalizumab-associated PML in MS is accompanied by the progression of clinical disability and imaging findings with contrast enhancement, expanding lesions with edema and mass effects (11, 12). The diagnosis of PML–IRIS has a crucial therapeutic relevance because treatment with corticosteroids, which is mostly associated with a better outcome, should not be initialized before any signs of immune reconstitution, since this may attenuate the specific T-cell response and lead to an unopposed progression of PML (13). Because there is still a lack of predictors of the onset and severity of PML–IRIS, clinicians may tend to withhold corticosteroids until a well-demonstrated IRIS response is identified (14). The occurrence of contrast enhancement is consistent with IRIS, but it is not a unique indicator with regard to the detection of PML–IRIS, since it is found in PML–IRIS as well as in early inflammation in the acute or asymptomatic disease stages of MS, and even simultaneously in PML (10, 15, 16).

Unlike other MR techniques, ^1^H magnetic resonance spectroscopy (^1^H-MRS) allows to characterize metabolic changes in pathological lesions as well as in normal-appearing brain tissue (17). However, ^1^H-MRS results regarding natalizumab-associated PML are limited, while more studies relate to HIV-associated PML (18, 19). In HIV-associated PML brain lesions, typical MRS findings are as follows: decreased levels of *N*-acetyl aspartate (NAA), increased Choline (Cho), elevated lactate and lipid, and variable myo-Inositol (mI) levels. Lesions of patients with HIV-associated PML–IRIS showed higher Cho/Creatine and mI/Creatine ratios, as well as increased lipid levels and reduced NAA/Creatine ratios than patients who did not have IRIS (18). There are a few case reports concerning MRS in MS patients with natalizumab-associated PML. In these studies, decreased NAA and increased Cho-levels were observed (20, 21). In a recent work by the authors the metabolic profiles in PML lesions of 15 MS patients in the post-PML setting have been explored confirming reduced NAA/Cr and NAA/Cho in early and late post-PML. Persisting JC virus findings more than 2 years after PML were associated with higher disability. Moreover, higher levels of lipid were detected in the early post-PML setting compared to late post-PML (22).

Therefore, the aims of this study were to investigate dynamic changes in ^1^H-MRS patterns in a larger cohort of MS patients suffering from natalizumab-associated progressive multifocal leukoencephalopathy at different disease stages, including the IRIS phase and the time between PML diagnosis and PML–IRIS and to examine whether ^1^H-MRS can provide additional paraclinical markers for the occurrence of PML–IRIS.

## Materials and Methods

### Patients

We included 20 RR-MS patients (12 females and 8 males) who were referred to our clinic with natalizumab-associated PML at different stages during the course of PML disease, and classified them into different PML status groups: pre-IRIS, IRIS, early post-PML, late post-PML. In 5 of these patients we were able to perform individual follow-up investigations. Most patients were referred to our institution for obtaining a second expert opinion at different disease stages. As a result, follow-up investigation could only be performed in MS patients treated in our institution for suspected PML or in early PML stages before the onset of IRIS. In some cases MRI examinations could not be performed during PML–IRIS because of the worsening of clinically affection requiring intensive care.

We performed MR imaging and ^1^H-MRS in 5 patients before the occurrence of PML–IRIS, one of these patients was scanned twice in the pre-IRIS period (pre-IRIS spectra *N* = 6) and in four patients during the occurrence of IRIS (IRIS spectra *N* = 4). In addition, 16 patients were observed after amelioration of the PML. Three of them received a follow-up examination (post-PML spectra *N* = 19). This patient group was further subdivided into two subgroups according to the time interval between IRIS and the time of MR examination. The early-post-PML group consists of 6 patients who were examined 12 [6–19] months (median [range]) after IRIS, including 2 follow-up scans (early post-PML spectra, *N* = 8). The late post-PML group consisted of 11 patients (including 1 patient who was scanned twice) who were scanned after 38 [23–67] months (median [range]). This subdivision aimed at assessing the changes of metabolic patterns in PML lesions by comparing the early-post-PML situation with the late-post-PML phase, which is often characterized by a clinical stabilization.

Progressive multifocal leukoencephalopathy–IRIS was defined by an inflammatory response resulting in clinical worsening of symptoms, which was associated with contrast enhancement on MRI in all patients of the IRIS group (23). The details of the PML diagnosis and disease course and the functional outcome of the study population have been reported recently (24). In brief, after the onset of neurological symptoms consistent with PML, the diagnosis was verified by JC-virus DNA detection in the cerebrospinal fluid (CSF) in 18 patients. In two patients, the CSF probes were initially JC-virus-negative, still the clinical presentation and MRI findings were indicative of PML (diagnostic accuracy “possible”) and no other differential diagnosis was found (25). One of these patients was tested virus positive in a follow-up puncture; in the other patient, a follow-up CSF probe was not available since the patient rejected the second puncture. CSF samples were analyzed for JC-virus DNA at the Department of Virology, University of Düsseldorf. The detection limit of the method was 10 copies/ml CSF (26). In all included participants IRIS associated with gadolinium enhancement in PML lesions was observed. After PML diagnosis, all patients received plasma exchange or immunoadsorption to remove natalizumab and supportive treatment with mefloquine and mirtazapine (9).

At each given MR examination, the clinical status was extracted from the patient files for each patient, including the Expanded Disability Status Scale (EDSS) and Karnofsky Performance Scale (KPS) (27, 28). The PML-related worsening of EDSS and KPS were defined as the EDSS and KPS difference between scoring 1 year before PML-diagnosis and scoring at the time of MR acquisition.

This non-interventional study was carried out in accordance with the recommendations of the ethics committee of the Ruhr-University Bochum, Germany (approval no. 4566-13). All subjects gave written informed consent in accordance with the Declaration of Helsinki.

### ^1^H-MRS and MR Imaging of PML Lesions

^1^H-MRS and MRI of the brain were performed on a single 3 T MRI System (Philips Achieva, Best, The Netherland) using a 32-channel head matrix coil. To localize the PML lesions and position of the volume of interest (VOI) for MRS, isotropic 3D sequences were performed, consisting of sagittal T1 fast field echo (repetition time/echo time TR/TE: 10/4.6 ms, inversion time TI: 1,000 ms, echo train length 164, matrix 240 × 240, 180 slices, resolution 1 mm × 1 mm × 1 mm) with and without Gadolinium administration, and sagittal fluid-attenuated inversion recovery (FLAIR) (TR/TE: 4800/291 ms, TI: 1650 ms, echo train length 182, matrix 240 × 240, 170 slices, resolution 1 mm × 1 mm × 1 mm). For ^1^H-MRS, a volume selective 2D-PRESS chemical shift imaging sequence (TR/TE: 2,000/45 ms, bandwidth 2,000 Hz, 1,024 spectral points, 128 measurements) was used to excite a brain region inside the skull of 82 mm × 90 mm × 15 mm dimension (left-right × anterior–posterior × head–feet dimension). Outer volume signal suppression by 10 circular saturation slices (30 mm thickness) which were oriented perpendicular to the excited plane was used in order to avoid signal distortions by susceptibility artifacts and interfering signals of subcutaneous fat.

Spectroscopic imaging was achieved by phase encoding in two directions within the excited plane to cover an axial field of view of 230 mm × 190 mm. The in-plane resolution was 10 mm × 10 mm × 15 mm. The preparation phase of ^1^H-MRS consisted of automatic procedures for water-suppression, shimming, and tuning of radiofrequency and gradient system, as well as an acquisition without water suppression for correction of magnetic field distortions.

The excited plane was positioned with the aim of covering PML lesions and normal appearing white matter (NAWM). If more than one region affected by PML lesions was visible on the FLAIR images, the excitation plane was positioned aiming to transect the largest lesion. In case of follow-up examinations, care was taken to choose identical anatomical locations and voxel sizes. Whenever possible, single MS lesions in white matter regions, which were apparently not affected by PML, were also covered.

Progressive multifocal leukoencephalopathy lesions were identified and differentiated from MS lesions by comparison with pre-existing, eventually external MRI scans acquired before the onset of PML by experienced raters (RS, CL). Differentiation was based on lesion location and morphology (subcortical, adjacent to U-fibers, diffuse demarcation to white matter), diffusion restriction (if diffusion weighted images were available) and patterns of Gadolinium uptake in the event of IRIS.

**Figure 1:**
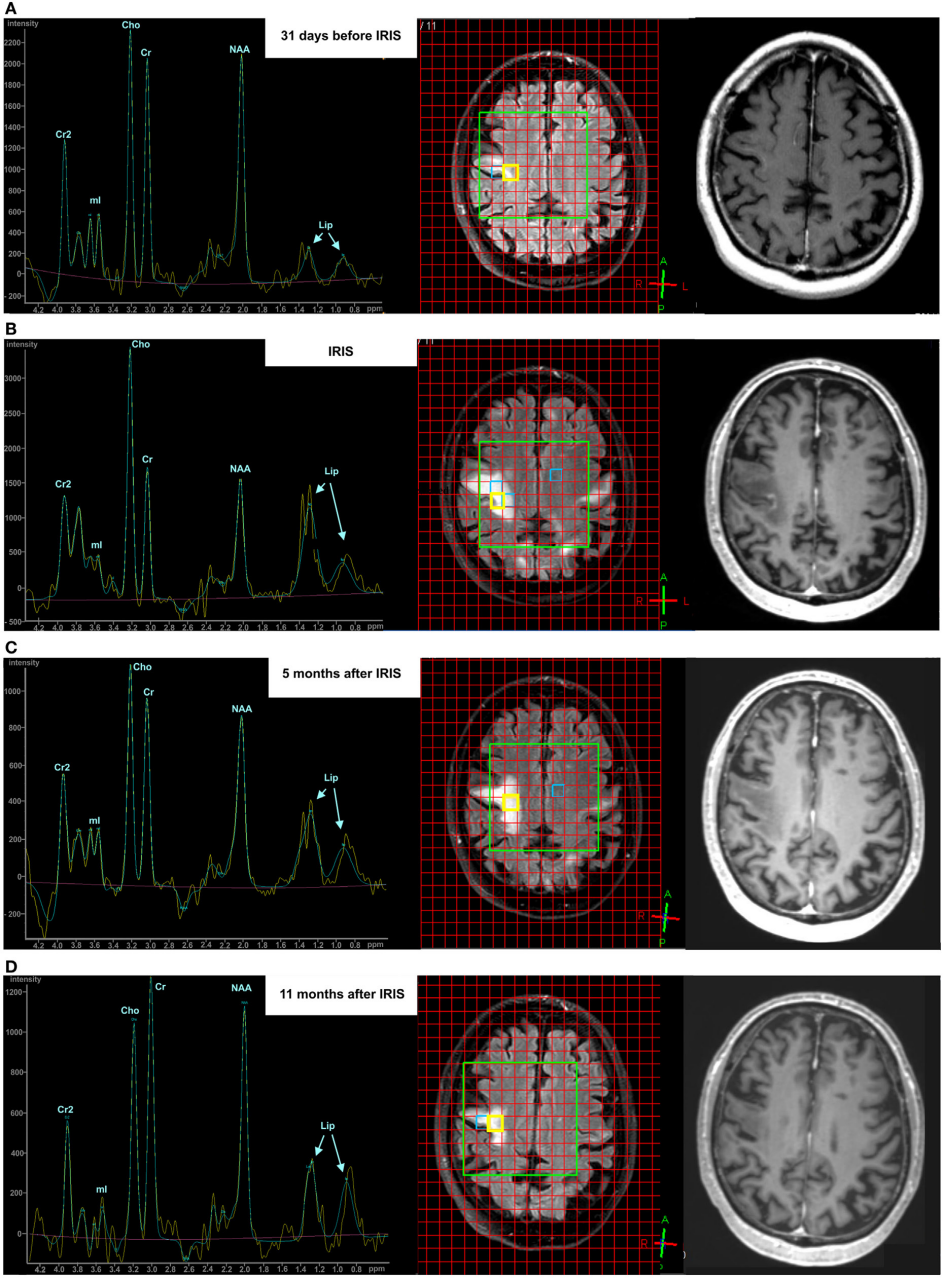
Exemplary course of progressive multifocal leukoencephalopathy (PML) in a single patient visualized by conventional magnetic resonance imaging (fluid-attenuated inversion recovery-sequence and Gadolinium-enhanced T1 sequence) and ^1^H-MRS during the pre-immune reconstitution inflammatory syndrome (IRIS) phase [**(A)** upper row], IRIS phase [**(B)** middle row], 5 months post-PML phase [**(C)** third row], and 11 months post-PML phase [**(D)** lower row].

Figure 1 demonstrates the course of changes in the metabolic patterns in a single patient (no. 1) in the pre-IRIS, IRIS, and two post-PML examinations (for details, see Results). The axial FLAIR images (middle column) illustrate typical positioning of the VOI (green box) covering PML lesions as well as NAWM. The evaluated spectra were based on selected single voxels (yellow box) corresponding to a volume of 1.5 ml. The PML spectra were positioned in the center of the lesions. In follow-up examinations, care was taken to choose identical evaluation planes and voxel positions. The corresponding contrast-enhanced T1 images (right column) show contrast uptake and evolution of T1 hypodensity at the location of the PML lesions.

### Spectral Processing

The vendor’s automatic software SpectroView was used for ^1^H-MRS signal post-processing (29, 30). The automatic processing included time domain filtering (Gaussian multiplication: 5 Hz and exponential multiplication: −3 Hz), residual water suppression by high-pass filtering (width 30 Hz) and fast Fourier transformation. By using prior knowledge of the expected metabolites, SpectroView quantified the frequency domain data by phase correction and the fitting of peaks. The baseline was fitted by a polynomial function and subtracted from the whole spectrum. Based on the peak integrals of the main metabolites NAA (2.0 ppm), Cr (3.1 ppm), Cho (3.2 ppm), mI (3.6 ppm), and lipid (Lip_0.9ppm_ and Lip_1.3ppm_, 0.9 and 1.3 ppm), metabolite ratios were calculated (NAA/Cr, NAA/Cho, Cho/Cr, and Lip/Cr). The acquired spectra without water suppression were used to calculate the peak integral of the unsuppressed water signal for each voxel. This spectral analysis covered metabolite ratios extracted separately in the center of PML lesions and in contralateral NAWM in each patient, and non-PML MS lesions whenever possible.

Metabolite ratios of peak integrals of single spectra are widely used to report results of brain spectroscopy, when no external reference for the determination of absolute metabolite concentrations is available, and allow for the comparison of results between different studies. The Cr peak integral is commonly chosen as the denominator in these ratios because Cr, being related to the intracellular energy states, seems to remain relatively stable in many pathological situations and in different brain regions. Consequently, variations of the Cr-based metabolite ratios are interpreted as changes of the metabolite in the numerator. However, the assumption of constant Cr levels seems not to be valid in certain pathologies, such as acute ischemic stroke (31), various primary brain tumors (32), and notably in MS lesions (33, 34). Thus, aiming to investigate changes of the single metabolites in different phases of the PML disease, we calculated ratios of the peak integrals in PML lesions related to the corresponding peak integral of the contralateral voxel of NAWM: NAA_PML_/NAA_NAWM_ (NAA_PML/NAWM_), Cr_PML_/Cr_NAWM_ (Cr_PML/NAWM_), Cho_PML_/Cho_NAWM_ (Cho_PML/NAWM_), Lip_PML_/Lip_NAWM_ (Lip_PML/NAWM_). We corrected these so-called “single metabolite ratios” for possible errors induced by imperfect contralateral positioning of the voxels by prior division of each metabolite’s peak integral by the corresponding peak integral of unsuppressed water. Bearing in mind that this procedure relies on the assumptions, that the water concentrations in PML lesion and NAWM are similar, and that the spectral patterns in NAWM are stable during the different PML phases, the results of these single metabolite ratios can only be interpreted as an estimation of the true courses of the single metabolite changes in PML lesions.

### Statistical Analysis

SPSS 22 (IBM SPSS, Chicago, IL, USA) was used for all statistical analyses. Due to the relatively small group sizes, we used non-parametric statistical tests. Group differences between the spectral results of PML lesions in the PML status groups, NAWM, and non-PML MS lesions were assessed by non-parametric Kruskal–Wallis tests. If these results were significant, SPSS 22 calculated *post hoc* pairwise comparisons corrected for multiple comparisons based on Dunn–Bonferroni tests (35). In addition, the group results of the metabolite ratios were visualized by box-plots (median, interquartile range, minimum, maximum).

Associations between MRS results in PML lesions and the time interval relative to IRIS in the entire patient group were assessed by Spearman correlation analyses. Correlations between disability parameters (KPS and EDSS) and metabolite ratios in PML lesions and single metabolite ratios (relating the peak integrals in PML lesion to contralateral NAWM) were also investigated by separate Spearman correlation analyses in the PML status subgroups.

## Results

### Demographic Data

Table 1 summarizes demographic data and clinical status at the time of ^1^H-MRS examination and one year before the occurrence of PML of all patients. Mean age, disease duration, and the number of natalizumab infusions before PML were (mean ± SD) 42 ± 10 years, 11.5 ± 5.5 years, 50 ± 19 infusions, respectively. It should be noted that if there were follow-up investigations available, one individual patient contributed ^1^H-MRS-results in different PML status groups.

**Table 1:** Demographic and clinical characteristics of participants.

Pat. no.	Age range at MRS (years)	Duration between MS diagnosis and PML (years)	No. of NAB^a^ infusions	PML group	Duration since IRIS (days)^b^	EDSS at MRS	EDSS at 1 year before PML	KPS at MRS	KPS at 1 year before PML
1	60–65	19	96	Pre-IRIS	−31	4.5	2	80	90
1				IRIS	0	6.5	2	40	90
1				E-post-PML	155	5.5	2	50	90
1				E-post-PML	340	5.5	2	50	90

2	40–45	23	55	E-post-PML	341	6.5	2	50	70
3	20–25	4	22	E-post-PML	372	2.5	2	90	90
4	26–30	6	49	E-post-PML	550	3.5	2	70	100
5	36–40	8	48	E-post-PML	589	1.5	1.5	100	100
6	40–45	6	48	L-post-PML	713	4	2	70	80

7	40–45	9	51	L-post-PML	806	5.5	3.5	60	80
7				L-post-PML	1,426	5	3.5	70	80

8	36–40	16	49	L-post-PML	837	4	2.5	67	90
9	30–35	11	62	L-post-PML	899	7.5	1	50	100
10	46–50	11	37	L-post-PML	1,178	4	2.5	80	90
11	50–55	17	40	L-post-PML	1,178	5	2	80	100
12	36–40	7	26	L-post-PML	1,395	6.5	3.5	60	70
13	30–35	9	30	L-post-PML	1,581	7	1.5	60	90
14	40–45	4	27	L-post-PML	1,736	5	2	40	100
15	36–40	12	34	L-post-PML	2,077	6.5	2	50	90

16	50–55	13	70	Pre-IRIS	−38	7	3.5	40	90
16		13		Pre-IRIS	−14	7.5	3.5	30	90

17	60–65	21	41	Pre-IRIS	−10	8	4	40	60

18	46–50	10	106	Pre-IRIS	−40	3.5	2.5	70	90
18		10		IRIS	0	4	2.5	60	90

19	40–45	9	82	Pre-IRIS	−30	3.5	2.5	80	90
19		9		IRIS	0	6.5	2.5	40	90
19		9		E-post-PML	120	5.5	2.5	60	90
19		9		E-post-PML	247	5	2.5	70	90

20	56–60	16	60	IRIS	0	4	4	60	90

*^a^NAB = Natalizumab*.

*^b^Negative values for time before IRIS*.

*MRS, MR spectroscopy; MS, multiple sclerosis; PML, progressive multifocal leukoencephalopathy; E-post-PML, early post PML; L-post-PML, late post-PML; EDSS, expanded disability severity scale; KPS, Karnofsky performance scale*.

### PML Lesion Characteristics

The individual PML lesion presentation in conventional MRI at the time of MRS compared to the situation at PML diagnosis and PML–IRIS was assessed. The details are provided in the Table S1 in Supplementary Material in the additional online material. All patients with supratentorial PML had typical MRI features with progressive PML lesion evolution after onset, large lesions in FLAIR imaging with typically subcortical location extending into the gyrus and U-fiber involvement. Three patients had infratentorial PML with brainstem and cerebellar involvement. Contrast enhancement has been observed in all patients at PML–IRIS. The PML lesions were hypointense on unenhanced T1 weighted images in most of the cases except three patients in the pre-IRIS and early IRIS phase. The majority of patients who received MRS examination in the post-PML phases showed considerable brain atrophy at the PML lesion locations. To characterize the PML lesions, a semi-quantitative MRI sum score was derived which describes the extent of the lesions and the affected brain region on FLAIR weighted imaging (range 1–9: 1 = focal lesions affecting one gyrus, 9 = bi-hemispheric lesions covering both hemispheres).

### ^1^H-MRS Findings

Figure 1 illustrates exemplarily the longitudinal brain metabolite changes during the course of the PML-disease in a PML lesion of a single patient (left column). The first MRS examination was acquired 3 weeks after PML diagnosis and immunoadsorption to remove natalizumab. FLAIR-weighted MR images depict the PML lesions and the localization of the spectra (middle column). The extension of the FLAIR lesions increased from the pre-IRIS phase until 5 months after PML–IRIS and was found decreased at 11 months after IRIS. Contrast-enhanced T1 weighted images (right column) showed subtle signs of contrast accumulation concordant with early inflammatory PML in the pre-IRIS phase, marked rim and punctuate enhancement in the IRIS phase, and increasing T1 hypointensity at the PML lesion location. In pre-IRIS PML lesion spectrum (Figure 1A) compared to the pooled NAWM results (Table 2), we saw reduced levels (>1 SD) of NAA/Cr and NAA/Cho (lesion vs. NAWM: NAA/Cr 1.17 vs. 1.50 ± 0.28; NAA/Cho 1.09 vs. 1.98 ± 0.57) and an increase in Lip/Cre which exceeded 2 SDs (lesion vs. NAWM: Lip/Cr 0.96 vs. 0.50 ± 0.21) in a location at which a large PML lesion arose only later on, in the IRIS and post-IRIS phase. In the IRIS phase (Figure 1B), marked increases of Cho/Cr and lipid were apparent as compared to the pre-IRIS phase at the same location. Cho/Cr and Lip/Cr decreased in the post-PML phases [(Figure 1C), 5 months after IRIS] and ceased even more 11 months after IRIS (D), although they were still elevated compared to the pre-IRIS spectrum. In the spectra acquired in the IRIS and early post-IRIS phase (Figures 1B,C), the rising of the lipid signals were dominated by the increase of the peak at 1.3 ppm (Lip_1.3ppm_) originating by protons in CH_2_ groups of fatty acids. Furthermore in this patient, a superimposition of lactate signal was visible in (Figures 1B,C) at this peak position.

**Table 2:** Results of ^1^H MRS: metabolite ratios in PML lesions subdivided into different PML phases, compared to NAWM and non-PML MS lesions (NAA/Cr, NAA/Cho, Cho/Cr, Lip/Cr) and single metabolite ratios of the results in a PML voxel divided by the result in the corresponding voxel in contralateral NAWM (NAA_PML/NAWM_, Cr_PML/NAWM_, Cho_PML/NAWM_, Lip_sum PML/NAWM_, Lip_1.3ppm PML/NAWM_, Lip_0.9ppm PML/NAWM_).

	PML lesions				NAWM	MS lesions	*p*^a^
	
Median [range]	Pre-IRIS	IRIS	Early post PML	Late post PML			
*N*	6	4	8	11	29	6	
NAA/Cr	0.96 [0.81–1.17] *p* = 0.003^b^ (*NAWM*)*p* = 0.018^b^ (*MS*)	1.2 [0.8–1.3]	1.0 [0.5–1.7] *p* = 0.012^b^ (*NAWM*)	1.2 [0.9–1.4]	1.5 [0.8–2.1]	1.5 [1.2–2.1]	<0.001
NAA/Cho	0.99 [0.79–1.14] *p* = 0.004^b^ (*NAWM*)	0.6 [0.4–0.7] *p* = 0.001^b^ (*NAWM*)	1.1 [0.5–2.5] *p* = 0.018^b^ (*NAWM*)	1.5 [0.9–2.0]	1.8 [1.2–3.2]	1.7 [1.0–2.5]	<0.001
Cho/Cr	1.05 [0.78–1.14]	2.1 [1.2–2.9] *p* = *0.003*^b^ (*NAWM*)	0.9 [0.7–1.3]	0.9 [0.7–1.2]	0.8 [0.5–1.3]	0.9 [0.7–1.3]	0.002
Lip/Cr	1.5 [0.5–3.4] *p* = 0.014^b^ (*NAWM*) *p* = 0.025^b^ (*L-post-PML*)	2.6 [1.2–4.2] *p* = 0.004^b^ (*NAWM*) *p* = 0.006^b^ (*L-post PML*)	1.1 [0.6–2.1] *p* = 0.007^b^ (*NAWM*) *p* = 0.017^b^ (*L-post PML*)	0.4 [0.1–1.0]	0.5 [0.2–1.1]	0.7 [0.5–1.7]	<0.001
NAA_PML/NAWM_	0.63 [0.54–0.99]	0.5 [0.3–0.6]	0.5 [0.3–0.9]	0.7 [0.5–1.1]			0.094
Cr_PML/NAWM_	1.19 [0.70–1.33] *p* = 0.111^b^(*E-post-PML*)	0.6 [0.4–1.0] *p* = 0.072^b^ (*pre-IRIS*)	0.8 [0.3–1.0]	0.9 [0.6–1.5]			0.037
Cho_PML/NAWM_	1.35 [0.96–1.61] *p* = 0.008^b^ (*E-post-PML*)	1.2 [1.1–1.7] *p* = 0.055^b^ (*E-post-PML*)	0.8 [0.6–1.2]	1.1 [0.5–1.9]			0.006
Lip_sum PML/NAWM_	2.64 [1.47–3.78]	3.1 [2.5–3.3] *p* = 0.039^b^ (*L-post-PML*)	2.0 [1.1–3.2]	1.5 [0.5–3.4]			0.028
Lip_1.3ppm PML/NAWM_	3.40 [2.49–5.25] *p* = 0.109^b^ (*L-post-PML*)	6.1 [2.7–10.2] *p* = 0.025^b^ (*L-post-PML*)	3.0 [1.3–7.2]	1.5 [0.7–3.5]			0.015
Lip_0.9 ppm PML/NAWM_	2.08 [1.30–2.68]	1.5 [1.1–3.8]	1.6 [0.2–3.2]	1.1 [0.2–4.5]			0.609

*^a^Significance p of differences between groups by Kruskal-Wallis tests*.

*^b^Significant post hoc pairwise comparisons, corrected for multiple comparisons by Dunn-Bonferroni correction*.

*NAWM, normal appearing white matter; MS, multiple sclerosis; PML, progressive multifocal leukoencephalopathy; IRIS, immune reconstitution inflammatory syndrome; NAA, N-acetyl aspartate; Cr, Creatine; Cho, Choline; Lip, Lipid; L-post-PML, late post-PML*.

**Figure 2:**
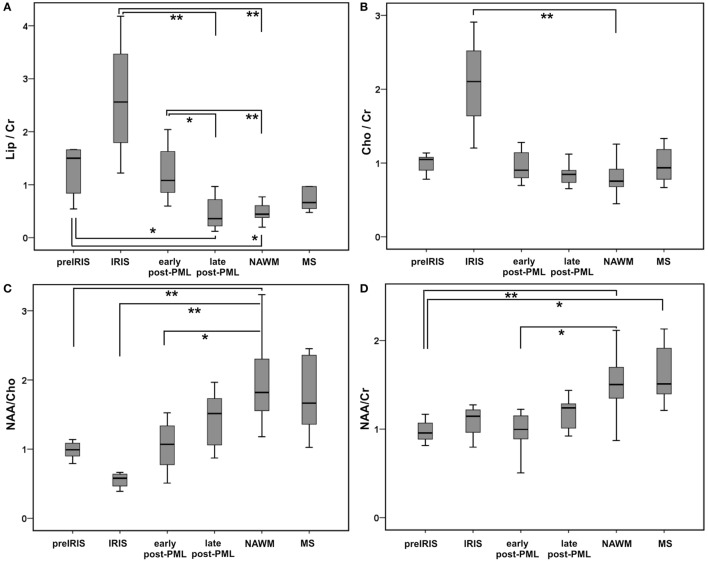
Qualitative group comparisons between different progressive multifocal leukoencephalopathy (PML) phases shown as boxplots for main metabolite ratios Lip_sum_/Cr **(A)**: upper row left panel, Cho/Cr **(B)**: upper row right panel, NAA/Cho **(C)**: lower row left panel, NAA/Cr **(D)**: lower row right panel. Boxes, interquartile range; line, median; and error bars, minimum–maximum; pre-immune reconstitution inflammatory syndrome (IRIS), IRIS, early post-PML, and late post-PML represent spectral results in PML lesions; normal appearing white matter (NAWM) and multiple sclerosis (MS) show pooled spectral results in NAWM and non-PML MS lesions. Significant group differences (pairwise testing after significant Kruskall–Wallis tests; corrected for multiple comparisons by Dunnett–Bonferroni tests) are marked by ** if *p* < 0.010, * if *p* < 0.050, (*) for trends: 0.050 ≤ *p* < 0.100.

**Figure 3:**
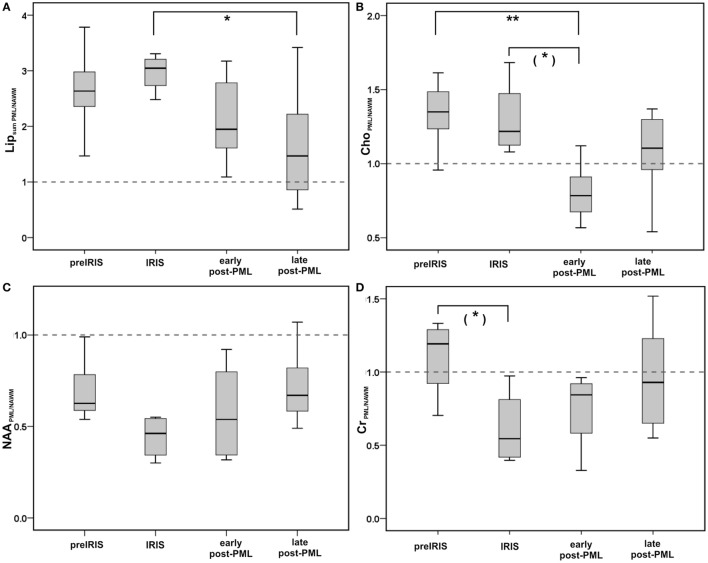
Group comparisons between different PML phases shown as boxplots for single metabolite ratios comparing PML lesions with contralateral NAWM for NAA_PML/NAWM_, and Lip_sum PML/NAWM_ **(A)**: upper row left panel, Cho_PML/NAWM_ **(B)**: upper row right panel, NAA_PML/NAWM_ **(C)**: lower row left panel, Cr_PML/NAWM_ **(D)**: lower row right panel. Boxes: interquartile range, line: median, error bars: minimum-maximum; Significant group differences (pairwise testing after significant Kruskall Wallis tests; corrected for multiple comparisons by Dunnett–Bonferroni tests) are marked by ** if *p* < 0.010, * if *p* < 0.050, (*) for trends: 0.050 ≤ *p* < 0.100.

Table 2 summarizes the ^1^H-MRS-results within the different patient groups regarding the integrals of metabolite peaks derived in the center of PML lesions at different PML phases and in NAWM and some (non-PML)-MS lesions. We compared to the metabolite ratios of the main metabolites (NAA, Cr, Cho, and Lipid) within voxels of PML lesions, NAWM, and non-PML-MS lesions. In addition, we regarded single metabolite ratios by calculating the ratios of the signal areas in PML lesion compared to NAWM voxels, in order to estimate the change of the single metabolites during different phases of PML. Figures 2 and 3 (boxplots) further illustrate these interrelations.

The Kruskal–Wallis tests showed highly significant differences between the subgroups for NAA/Cr, NAA/Cho, Cho/Cr, and Lip/Cr. *Post hoc* pairwise comparisons showed significant elevations of Lip/Cr at the PML lesion locations in the pre-IRIS, IRIS, and the early post-PML phases relative to NAWM and relative to PML lesions in the late post-PML setting. These Lip/Cr increases were highest in the IRIS phase. In line, NAA/Cho was reduced in PML lesions in the pre-IRIS, IRIS (most reduced), and early post-PML phases compared to NAWM.

Cho/Cr was considerably increased relative to NAWM exclusively in the IRIS phase. By contrast, NAA/Cr was constantly reduced in all PML phases compared to NAWM and MS lesions, reaching statistically significance compared to NAWM in pre-IRIS and early post-PML lesions. Thus, Lip/Cr and Cho/Cr seemed to be the most promising dynamic metabolite ratios that were related to the onset of the IRIS phase in PML. Figure 2 further illustrates these interrelations.

The single metabolite ratios of PML lesions relative to the contralateral NAWM voxel (Table 2; Figure 3) showed reduced levels (<1.0) of NAA_PML/NAWM_ in all PML phases, which were lowest in the IRIS group, while the differences between the PML groups were not significant (Figure 3C).

Creatine (Figure 3D): compared to the pre-IRIS phase, in which Creatine_PML/NAWM_ was elevated (>1.0), we observed a significant reduction of the Cr_PML/NAWM_ level in the IRIS group, and increasing Cr_PML/NAWM_ levels in the post-PML phases reaching close-to-normal levels in the late post-PML group.

Choline (Figure 3B): we observed markedly elevated Cho_PML/NAWM_ levels in pre-IRIS and IRIS patients without significant differences between these groups. By contrast, in the early post-PML group the Cho_PML/NAWM_ ratio was significantly lower (<1.0) than in the pre-IRIS and IRIS groups.

Lipid (Figure 3A): the single metabolite ratios of the summed lipid signal in PML compared to NAWM were high in pre-IRIS and highest (> = 3) in the IRIS group. Lip_sum PML/NAWM_ was lower, but still considerably elevated compared to NAWM in early post-PML. Also in late post-PML patients, the Lip_sum PML/NAWM_ ratio was higher than normal; the differences in comparison to the IRIS patients were highly significant in this group.

In Table 2, we additionally analyzed the lipid contributions of the peaks at 0.9 ppm (CH_3_ groups) and 1.3 ppm (CH_2_-groups) separately. We found that the increase of the summed lipid signal was dominated by Lip at 1.3 ppm. The levels in pre-IRIS and IRIS were markedly elevated in Lip_1.3ppm_ _PML/NAWM_ (≥6; highly significant in comparison to the late post-PML setting), reaching up to 4-fold values as compared to Lip_0.9ppm PML/NAWM_ in the IRIS phase.

### Correlation Analysis of ^1^H-MRS Metabolite Ratios in PML Lesions and Time Difference to IRIS

**Figure 4:**
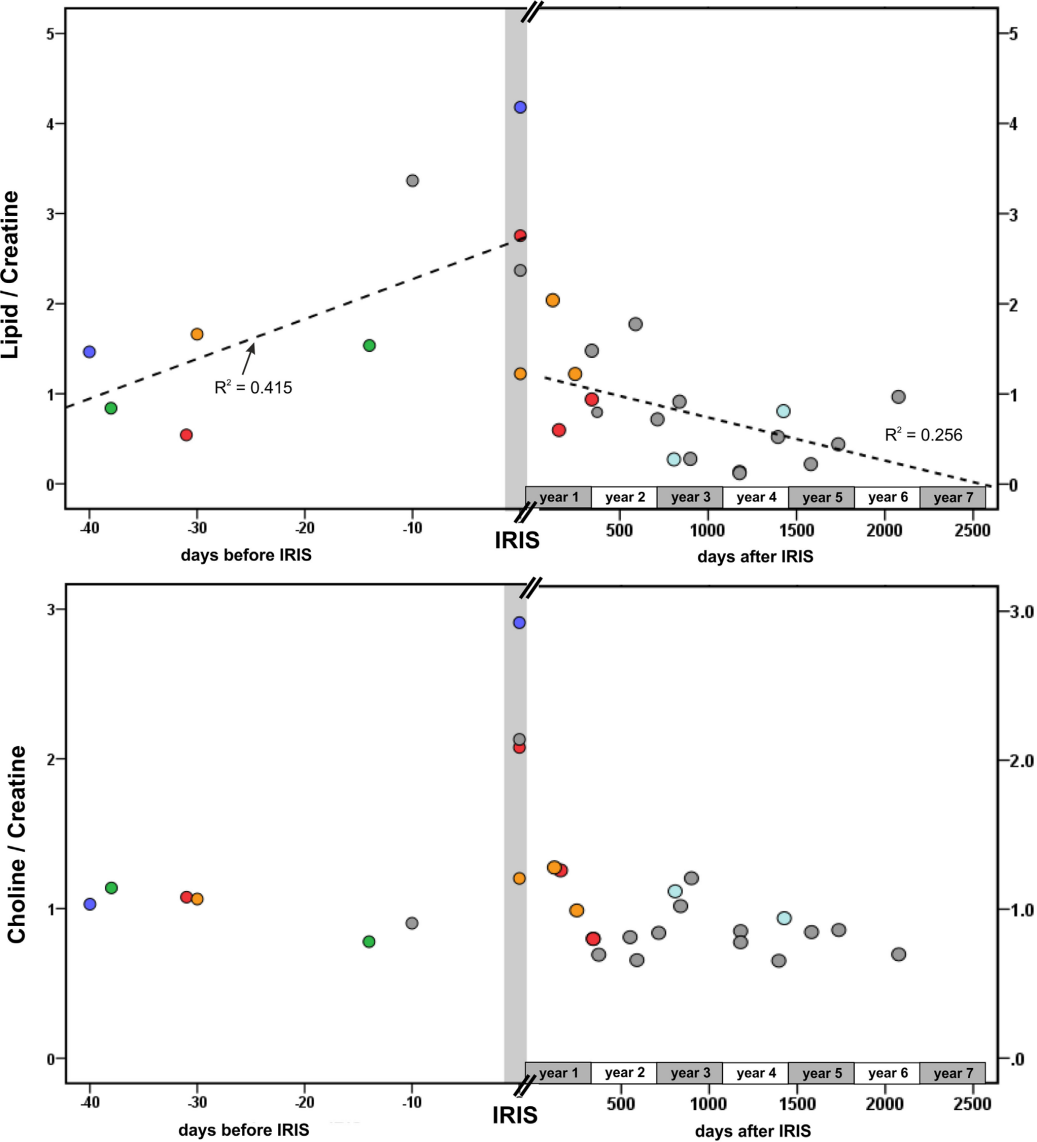
Dependence of Lip/Cr and Cho/Cr on the time difference between the MRS examination and the onset of IRIS. Gray symbols: all patients who had a single MRS examination; red symbols, patient no. 1; green symbols, patient no. 16; blue symbols, patient no. 18; orange symbols, patient no. 19 (here: IRIS = early IRIS with beginning of contrast enhancement), light blue symbols, patient 7. Dotted lines: linear regression of Lip/Cr and time difference before (*R*^2^ = 0.415) and after IRIS (*R*^2^ = 0.256).

Spearman correlation analysis demonstrated a significant steady increase of Lip/Cr depending on the time period before the occurrence of IRIS (ρ = 0.619, *p* = 0.028). Furthermore, in the post-IRIS phase, Lip/Cr levels were significantly negative correlated with the time difference after IRIS (ρ = −0.518, *p* = 0.014), indicating a slow decline to normal levels. In contrast to Lip/Cr, the levels of Cho/Cr were elevated uniquely at the time of IRIS and were normal in the pre- and post-IRIS phases. Figure 4 illustrates these correlations; patients who received follow-up examinations are marked by five different colors. These associations were also reflected by the individual longitudinal courses of Lip/Cr and Cho/Cr in three of the four patients who had follow-up examinations in the pre-IRIS and IRIS phase. In one patient who showed no marked increases of Lip/Cr or Cho/Cr in the PML lesion at IRIS (no. 19, orange symbols in Figure 4), MRS was acquired at the beginning of PML–IRIS, which was defined by subtle signs of Gadolinium enhancement in the PML lesion. The full clinical IRIS manifestation with considerable increase of gadolinium enhancement in MRI was observed 2 weeks after the MRS. For this reason, Lip/Cr and especially Cho/Cr increases might have been missed by the MRS assigned to the IRIS phase in this patient.

Furthermore, when regarding the single metabolite ratios of PML lesions compared to contralateral NAWM we observed significant negative correlations of NAA_PML/NAWM_ and Cr_PML/NAWM_ in the pre-IRIS and IRIS phase (ρ = −0.825, *p* = 0.002, and ρ = −0.732, *p* = 0.008) with time difference before IRIS, indicating a steady decrease of the NAA_PML/NAWM_ and Cr_PML/NAWM_ in that phase of the disease. The decrease of the NAA_PML/NAWM_ and Cr_PML/NAWM_ levels during the time between the PML diagnosis and occurrence of PML–IRIS was also found in the individual follow-up examinations. In the post-IRIS phase, there were no significant correlations of the single metabolite ratios with the time after PML–IRIS. Details of this analysis are illustrated in the Figures S1 and S2 in Supplementary Material.

### Correlation Analysis of ^1^H-MRS Metabolite Ratios and Disability Status

All PML patients clinically worsened indicated by a marked EDSS increase and a KPS decrease within 1-year interval before PML and during pre-IRIS/IRIS. In the post-PML period, the patients recovered (24) as compared to IRIS.

To investigate associations between the ^1^H-metabolite ratios and clinical disability, the differences of KPS and EDSS between the time point of ^1^H-MRS and 1 year before PML were analyzed. Pre-IRIS and IRIS phases (raising Lip/Cr and Cho/Cr and decreasing NAA/Cho levels) and the post-PML phases (decreasing Lip/Cr and Cho/Cr, recovery of NAA/Cho) were analyzed separately.

**Figure 5:**
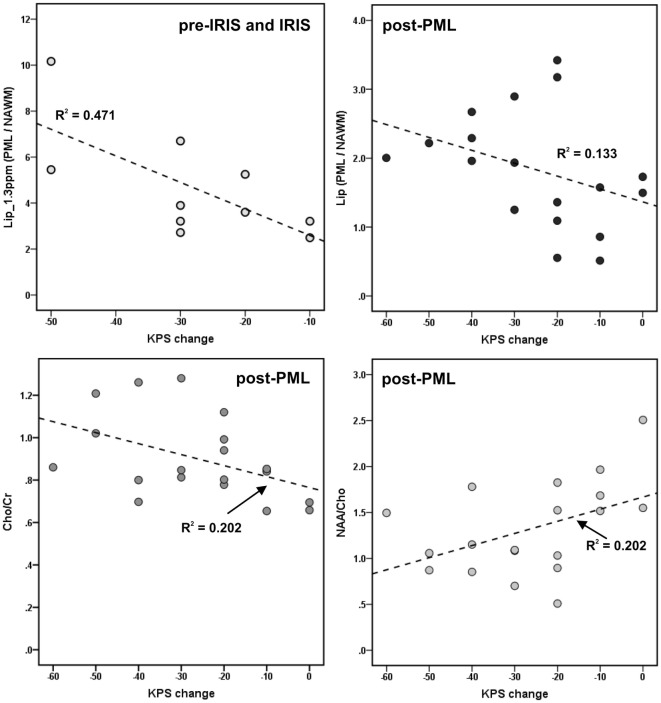
Associations between metabolite results and progressive multifocal leukoencephalopathy (PML)-related disability [change of Karnofsky Performance Scale (KPS) between time of MRS and 1 year before PML] in the pre-immune reconstitution inflammatory syndrome (IRIS) and IRIS phase, and in the post-PML phase; only significant correlations are depicted. Dotted lines and *R*^2^: linear regression line and coefficients of determination.

In the post-PML group (*N* = 19), Spearman correlation analyses showed a significant positive correlation between NAA/Cho in PML lesions and the KPS change (ρ = 0.481, *p* = 0.018), significant negative correlations between Cho/Cr and the KPS change (ρ = −0.505, *p* = 0.014) and Lip_sum PML/NAWM_ and KPS change (ρ = −0.488, *p* = 0.020). In the pre IRIS and IRIS patients (*N* = 10), the increase of the lipid peak at 1.3 ppm in PML lesions relative to NAWM, Lip_1.3ppm PML/NAWM_, was significantly negative correlated with the KPS change (ρ = −0.669, *p* = 0.017). Figure 5 illustrates these associations. No other significant correlations of the KPS or the EDSS were observed in any subgroup.

## Discussion

In this explorative ^1^H-MRS study, characteristic metabolic changes in brain lesions of 20 MS patients were examined at different stages during the course of natalizumab-associated PML. The differences of the metabolic patterns between different PML stages in the cross-sectional investigations were supported by concordant results of the longitudinal course in follow-up examinations of five PML patients.

### Metabolic Changes during PML–IRIS Phase

Lipid and Choline resonance normalized to Creatine were the most promising metabolic markers for detecting the onset of the IRIS phase in the course of the PML disease: while Lip/Cr increased before the occurrence of IRIS with a maximum manifestation during the PML–IRIS phase, marked rises of Cho/Cr were found exclusively during the IRIS phase. Significant negative correlations between Lip/Cr and the number of days before IRIS and between Lip/Cr and the time after IRIS additionally confirmed the clinical relevance of the Lip/Cr ratio. Several days before PML–IRIS, the Lip/Cr ratio already seems to increase continuously, which could be helpful in detecting the onset of PML IRIS with the consequence of the initiation of a standardized recommended therapy regime (9).

The additional estimation of the changes of the individual metabolite signals throughout the different PML phases by assessment of the ratios in the PML lesions compared to the contralateral NAWM voxel indicated a decrease of Cr from slightly above-normal levels in the pre-IRIS to markedly sub-normal levels in the IRIS phase. This drop of the Cr level probably contributed to the observed changes of Lip/Cr and Cho/Cr. Lip_sum PML/NAWM_ showed steadily increasing levels with time to IRIS onset, which was accentuated by Lip/Cr. Cho_PML/NAWM_ was similarly increased in pre-IRIS and in IRIS, so the Cho/Cr ratio may emphasizes the change due to reduced Cr_PML/NAWM_ especially during PML–IRIS. For the first time, these effects were demonstrated cross-sectionally, but also in follow-up examinations.

The metabolic changes relating to lipid and choline levels were probably evoked by the progressive inflammation-situation that has already been seen in HIV-related PML (18). *In vitro* studies demonstrated an association between MRS-visible lipids and the presence of activated immune cells, especially T cells (36), proposing the role of lipids as a marker for activated lymphocytes (37). Furthermore, the release of mobile lipids consisting of triglycerides and cholesterol esters was interpreted as an indicator for increased membrane fluidity, which may facilitate the migration of T cells (37). To gain further information about the metabolic pattern of lipids, we regarded the different resonances at 0.9 ppm (CH_3_ methyl groups in fatty acids) and 1.3 ppm (CH_2_ methylene groups). The total lipid peak (Lip sum_PML/NAWM_), which was increased in pre-IRIS and IRIS, was dominated by the marked increase of the peak at 1.3 ppm. Due to the chemical structure of fatty acids the proportion of CH2 to CH3 groups should lead up to eight-fold higher signals at 1.3 ppm than at 0.9 ppm, if lipids were the only source of the signal in this frequency range (38). The relatively high ratios of the signals at 1.3 ppm compared to 0.9 ppm (up to fourfold) indicate a considerable fraction of free lipid production as a source of this signal increase at IRIS. This could be partly influenced by overlaid lactate signal, which may be attributed to anaerobic metabolism and activated glycolysis in macrophages (39). Considering the fact that the 0.9 ppm resonance was also significantly elevated compared to the post-PML setting, the presence of mobile lipids and pathologically altered macromolecules should be taken into consideration as sources of these signal increases (40). In a previous study, the elevation of the resonances at 0.9 and 1.3 ppm was significantly higher in acute MS lesions than in chronic MS lesions. These signals have been interpreted as free lipids, and increased macromolecules contributing to both resonances, which were conceivably interpreted as biochemical markers of myelin fragments (38). We hypothesize that this interpretation might be transferable to acute PML (pre-IRIS and IRIS) in order to distinguish it from the post PML setting, which would provide useful additional diagnostic information in this context. In summary, it can be stated that elevated lipid resonances could be influenced by several conditions due to acute inflammation, like infiltration of activated T cells into the brain tissue, and varying proportions caused by overlaid lactate as well as demyelination inducing the detectability of macromolecular fragments.

### Contrast Enhancement and Metabolic Changes during PML–IRIS Phase

The specificity of MRI contrast enhancement for the onset of IRIS is limited, especially with respect to milder inflammatory PML (23) and the absence of enhancement should not preclude clinical consideration of IRIS (10, 15, 16). Therefore, the observed increase of the Lip/Cr ratio in PML lesions before IRIS by ^1^H-MRS can provide useful additional information and an early marker for the detection of PML–IRIS. Especially in case of asymptomatic PML patients, early diagnosis before onset of symptoms is very important concerning survival and clinical outcome (4).

In HIV-associated PML–IRIS, the increase in Cho/Cr ratio, which is interpreted as an indicator for demyelination and membrane turnover, was associated with MR evidence of contrast enhancement, whereas increased Lip/Cr ratios were independent of contrast enhancement (18). The present results confirmed these findings in natalizumab-related PML in MS, in which the flashback of the immune system and, thus, IRIS is usually more pronounced than in HIV–PML. An elevated Cho/Cr ratio was merely seen in the IRIS phase during contrast enhancement of the PML-lesion. Increased Cho/Cr ratios were found in other demyelinating and inflammatory conditions, in the context of increased membrane turnover, emphasizing the role of Choline as a component of the cell membrane (41). Therefore, evaluation of the combined pattern of the Lip/Cr and Cho/Cr ratios may be helpful to characterize the onset and course of the IRIS phase.

### Metabolic Changes Regarding Neuroaxonal Integrity during the Course of PML

The NAA/Cr ratio, which is a characteristic metabolic marker for neuroaxonal integrity (42), was reduced in PML lesions compared to NAWM in the pre-IRIS and early post-PML group, but was not significantly different when comparing the different PML phases. We interpret this finding by early and long-lasting axonal loss in PML lesions compared to NAWM. In the IRIS group, we observed a non-significant increase of NAA/Cr. By contrast, in HIV-associated PML-lesions, the NAA/Cr ratio was described to be decreased in PML–IRIS but was classified as less specific based on most diverse inflammatory processes resulting in neuronal or axonal damage (18). When regarding the single metabolite ratios, we found a reduction of both NAA_PML/NAWM_ and Cr_PML/NAWM_ during the IRIS phase. The significant negative correlations relating the decrease of NAA_PML/NAWM_ and Cr_PML/NAWM_ in the pre-IRIS and IRIS phase with time between the PML diagnosis and occurrence of PML–IRIS indicated a continuous reduction of NAA and Cr before IRIS, which was confirmed by the follow-up investigations. These simultaneous decreases probably explain the subsequent, relative constant levels of NAA/Cr.

However, in PML–IRIS, the interpretation of a reduction of Cr is speculatively regarding the drawback of the small patient number in this group. Reduced Cr levels compared to NAWM have been reported in chronic MS lesions (34). But still, increases of Cr during inflammatory CNS demyelination have been described in RR-MS (43, 44). These were interpreted as higher cell densities per spectroscopic VOI in glial structures, provided that neuroaxonal structures, which harbor Cr for their cellular homeostasis and energy supply, are still intact (45). By implication, in PML–IRIS the reduction of Cr could be interpreted as lower cell density and loss of intact neuroaxonal structures due to fatal cell break-down after a regulatory increase of Cr_PML/NAWM_ in pre-IRIS caused by inflammation in the presence of still-intact neuroaxonal structures. The return of Cr_PML/NAWM_ to normal values in chronic states may reflect regeneration processes driven by oligodendrocyte migration and astrocytic or oligodendrocytic remyelinating processes (44).

Considering the metabolic changes of NAA/Cho during the course of the disease, we showed dynamic changes with a reduced NAA/Cho ratio in pre-IRIS and a maximal decrease in PML–IRIS followed by restitution in the post-PML setting to level of NAWM. According to literature, the increase of Cho outweighs the decrease of NAA in new PML lesions compared to older lesions. We suggest that demyelinating processes may dominate neuronal loss in active PML lesions, while neuroaxonal loss is probably the main effect in older PML lesions (19). Regarding the single metabolite ratios of Cho_PML/NAWM_, this interpretation was supported since Choline was elevated in pre-IRIS and IRIS compared to early-post-PML, reflecting dynamic changes in an ongoing disease activity. Thus, NAA/Cho may reflect dynamic metabolic changes during the course of PML disease but is less suitable for the detection of the onset of IRIS as it combines two different pathological processes leading to opposite effects on the metabolite ratio.

### Association between Metabolite Patterns and Clinical Parameters

As far as we know, the association between ^1^H-MRS-acquired metabolite patterns and clinical parameters in MS patients at post-IRIS stages of natalizumab-associated PML have only been analyzed the authors’ own previous work (22). In this study, the therein reported associations between Cho/Cr and the PML-related disability burden were confirmed in a larger patient group. Correlations between Cho/Cr, NAA/Cho and Lip_sumPML/NAWM_ and KPS change in post-PML patients, associating the worse clinical outcome of PML survivors with persistently high Cho/Cr, low NAA/Cho, and high lipid levels were demonstrated. We suppose that Cho/Cr and Lip_sumPML/NAWM_, reflecting increased membrane turnover in demyelinating and inflammatory conditions, may correspond to more severe clinical affection because of ongoing inflammatory activity in PML lesions.

Moreover, NAA/Cho, representing a dynamic metabolic marker, seems to have clinical relevance in the post-PML setting. Persistent clinical disability may be a result of ongoing neuroaxonal degeneration and demyelination, reflected by low NAA/Cho levels.

In the pre-IRIS and IRIS groups, the additive analysis of the lipid resonance at 1.3 ppm, which was significantly increased in PML–IRIS showed a negative correlation with KPS change reflecting that higher lipid levels at 1.3 ppm were associated with a worse clinical status in acute PML. The correlation of the summed Lip_PML/NAWM_ in the summarized post-PML group with KPS-change could reflect the persistent presence of lipids in later PML stages as a possible marker of reduced clinical convalescence.

### Limitations

A few limitations of this survey need to be mentioned. The main limitation is the small number of included patients for follow-up MRS in the pre-IRIS and IRIS group characterizing the explorative manner of this study. Furthermore, since most of the included patients received only one MRS examination the probable temporal course of the metabolic changes related to PML was mostly deduced from these cross-sectional results, although the conclusions were strongly supported by evidence from concording follow-up examinations of five patients. As such, the herein presented preliminary results may differ from longitudinal examination of individual patients and should, therefore, be interpreted with caution.

Generally, it has to be stated that acquiring a large number of homogeneous longitudinal MR spectroscopic and imaging data on PML patients is a difficult task for several reasons: first, MRS is time-consuming (acquisition about 10 min.) and demands experienced users. Longitudinal MRS examinations have to be performed at a single scanner using a homogeneous scan protocol to ensure reproducibility of these examinations. Thus, patients have to be enrolled for suspected or early PML before the onset of IRIS and followed-up over a longer period in a single center. Since PML–IRIS is often accompanied by massive clinical worsening, requiring intensive care, these circumstances often prohibit prolonged MR scanning including MRS.

Another limitation is the lack of absolute metabolite quantification in this study. Apart from the presentation of metabolite ratios, we could only provide an estimation of the changes of the single metabolites by comparing the results in PML voxels with contralateral NAWM. These single metabolite ratios can be biased by occult disease related changes within the NAWM and by imperfect contralateral voxel positioning. Future studies should include absolute metabolite quantification techniques providing independent external concentration references. Furthermore, in the context of PML–IRIS, dedicated procedures for quantification of lipids and distinction from macromolecules and lactate, such as the metabolite nulling technique (38), should be used.

Natalizumab-associated PML is a rare complication of MS treatment. Thus, it is not probable that a single academic MS center will treat and follow-up many patients living in the nearby region for suspected PML in a reasonable time period. Instead, most patients will be referred to the center in the acute onset-phase of PML or for a second opinion and will be treated longitudinally in their home area. Others will be referred to the center in the post-PML setting for decision about reinitiation of an immunomodulatory MS treatment.

In future studies, common efforts of several MS centers using identical scanning protocols for imaging and MR spectroscopy would be desirable in order to gain longitudinal information about the temporal dynamics of brain metabolic MRS changes during the course of PML on a large number of patients.

In summary, our exploratory study provided indications that ^1^H-MRS can be a helpful tool to detect characteristic dynamic changes with clinical relevance during the course of natalizumab-associated PML in MS patients. The onset of PML–IRIS, in particular, was associated with rising Lip/Cr and Lipid resonance at 1.3 ppm and elevated Cho/Cr levels.

## Ethics Statement

This non-interventional stdy was carried out in accordance with the ethical standards laid down in the 1964 Declaration of Helsinki. The protocol was approved by the ethics committee of the Ruhr-University Bochum (approval no. 4566-13). Written informed consent was obtained from all participants prior to inclusion in the study.

## Author Contributions

RS: design of the work, acquisition, analysis, and interpretation of data for the work; drafting and critical revision of the work for important intellectual content; and final approval of the version to be published and agreement to be accountable for all aspects of the work. BB: acquisition, analysis, and interpretation of data for the work; interpretation of data for the work; drafting and critical revision of the work for important intellectual content; and final approval of the version to be published and agreement to be accountable for all aspects of the work. RH: acquisition and analysis of data for the work; critical revision of the work for important intellectual content; and final approval of the version to be published and agreement to be accountable for all aspects of the work. RG: conception of the work, interpretation of data for the work; critical revision of the work for important intellectual content; and final approval of the version to be published and agreement to be accountable for all aspects of the work. CL: conception and design of the work, interpretation of data for the work; critical revision of the work for important intellectual content; and final approval of the version to be published and agreement to be accountable for all aspects of the work.

## Conflict of Interest Statement

The authors declare that there was no conflict of interest related to this research. RS declares that there is no conflict of interests. BB has received speaker’s honoraria and research grant support from Bayer Healthcare. RH received research and travel grants from Biogen Idec and Novartis. GE received speakers or scientific grant support from BiogenIdec, TEVA Pharma, Bayer Healthcare, Genzyme, Almirall, and Novartis Pharma. RG received speaker’s and board honoraria from Biogen Idec, Baxter, Bayer Schering, Chugai Pharmaceuticals, Merck Serono, Novartis, Roche, Sanofi-Aventis, Talecris, and TEVA; received scientific grant support from Biogen Idec, Bayer Schering, Genzyme, Merck Serono, Novartis, and TEVA; serves as editor for Therapeutic Advances in Neurological Diseases and on the editorial boards of the American Journal of Pathology and the Journal of Neuroimmunology. CL received consulting and speaker’s honoraria from BiogenIdec, Bayer Schering, Novartis, Sanofi, Genzyme, and TEVA; has received research scientific grant support from Bayer Schering, TEVA, and MerckSerono; holds an endowed professorship supported by the Novartis Foundation.

## Abbreviations

PML, progressive multifocal leukoencephalopathy; JCV, John Cunningham Virus; HIV, human immunodeficiency virus; RR-MS, relapsing–remitting multiple sclerosis; MRI, magnetic resonance imaging; IRIS, immune reconstitution inflammatory syndrome; ^1^H-MRS, ^1^H magnetic resonance spectroscopy; NAA, *N*-acetyl aspartate; Cho, Choline; Cre, Creatine, Lip, Lipid, mI, myo-Inositol; CSF, cerebrospinal fluid; DNA, desoxyribonucleic acid; EDSS, Expanded Disability Status Scale; KPS, Karnofsky Performance Scale; TR, repetition time; TE, echo time; TI, inversion time; VOI, volume of interest; NAWM, normal appearing white matter.
