# Supplementary material for: Insight into Metabolic 1H-MRS Changes in Natalizumab Induced Progressive Multifocal Leukoencephalopathy Brain Lesions
Source: Front Neurol. 2017 Sep 5;8:454. doi: 10.3389/fneur.2017.00454 (PMC5591840; doi:10.3389/fneur.2017.00454)

Supplementary Material

Insight into metabolic ^{1}H-MRS changes in natalizumab induced progressive multifocal leukoencephalopathy (PML) brain lesions

Ruth Schneider, Barbara Bellenberg, Robert Hoepner, Gisa Ellrichmann, Ralf Gold, Carsten Lukas[*]

*** Correspondence:** Carsten Lukas, MD e-mail: carsten.lukas@rub.de

1 Supplementary Figures and Tables

Supplementary table 1: PML lesion characteristics in MRI at the time of MRS compared to the situation at PML diagnosis and at PML-IRIS

Pat. No.	PML group (disease duration)[a]	PML lesion score[b]	lesion distribution at MRS, compared to PML onset	affected region on FLAIR at MRS	Hypo-intense on T1 at MRS	CE at MRS	CE at IRIS	brain atrophy at PML lesion	location of spectrum
1	pre-IRIS (-31 d.)	1	single gyrus	frontal lobe; adjacent to right motor cortex	no	no		no	suspected PML lesion, right frontal lobe
1	IRIS (0 d.)	4	multilobar, increasing extension compared to preceeding MRS	frontal lobe; right and adjacent to left motor cortex	yes	yes	yes, punctate & rim enhancement	no	PML lesion, right frontal lobe
1	E- post-PML (5 m.)	4	multilobar, stable	frontal lobe; right and adjacent to left motor cortex	yes	yes		no	PML lesion, right frontal lobe
1	E- post-PML (11 m.)	4	multilobar, decreasing extension compared to preceeding MRS	frontal lobe; adjacent to right motor cortex	yes	no		no	PML lesion, right frontal lobe

2	E- post-PML (11 m.)	4	multilobar, increasing extension	left hemispheric frontal lobe & occipital lobe	yes	no	yes, punctate	yes	PML lesion, left frontal lobe
3	E -post-PML (12 m.)	6	multilobar, unknown	frontal lobe both hemispheres, left occipital lobe	yes	no	yes, punctate& patchy	no	PML lesion, left frontal lobe
4	E- post-PML (18 m.)	4	multilobar, increasing extension	frontal & parietal lobe, right hemisphere	yes	no	yes, punctate& patchy	yes, marked atrophy	PML lesion, right parietal lobe
5	E- post-PML (19 m.)	1	single gyrus, decreasing extension	frontal lobe, right hemisphere	yes	no	yes, punctate& patchy	no	PML lesion, left frontal lobe
6	L- post-PML (24 m.)	4	multilobar, increasing extension	temporal & occipital lobe, left hemisphere	yes	no	yes, patchy	yes	PML lesion, left occipital lobe
7	L- post-PML (26 m.)	8	multilobar, decreasing extension	frontal lobes widespread, both hemispheres	yes	no	yes, punctate	yes, marked atrophy	PML lesion, left frontal lobe
7	L- post-PML (46 m.)	6	multilobar, stable compared to preceeding MRS	frontal lobes widespread, both hemispheres	yes	no	yes, punctate	yes, marked atrophy	PML lesion, left frontal lobe
8	L- post-PML (27 m.)	7	right hemisphere,	frontal, occipital and temporal lobes, left	yes	no	yes, punctate	yes, marked	PML lesion left

			decreasing extension	hemispere				atrophy	occipital lobe
9	L- post-PML (29 m.)	4	multilobar, decreasing extension	cerebellum & brainstem, left hemisphere	yes	no	yes, diffuse & punctate	no	PML lesion brainstem left
10	L- post-PML (38 m.)	3	unilobar, decreasing extension	occipital right hemisphere,	yes	no	yes, diffuse	no	PML lesion right occipital lobe
11	L- post-PML (38 m.)	6	multilobar, decreasing extension	frontal, right & left hemisphere	yes	no	yes, diffuse & punctate	yes, marked atrophy	PML lesion, left frontal lobe
12	L- post-PML (45 m.)	9	multilobar, decreasing extension	frontal lobe adjacent to motoric areas & parietal lobe, left hemisphere	yes	no	yes; diffuse rim	yes	PML lesion, left frontal lobe
13	L- post-PML (51m.)	9	right hemisphere, decreasing extension	frontal, parietal & temporal lobe, right hemisphere;	yes	no	yes, unknown pattern	yes, marked atrophy	PML lesion, right frontal lobe;
14	L- post-PML (56 m.)	6	multilobar, both hemispheres, increasing extension	frontal & parietal lobes, right hemisphere more affected than left	yes	no	yes, homogeneous rim enhancement	yes	PML lesion, right frontal lobe;
15	L- post-PML (67 m.)	5	multilobar, decreasing	frontal & parietal lobe, left hemisphere	yes	no	yes, unknown	yes	PML lesion, left parietal lobe

			extension				pattern		
16	pre-IRIS (-38 d.)	6	multilobar, inceasing extension	frontal lobes in both hemispheres, temporal & occipital right hemisphere	yes	no	yes, punctate& patchy, rim enhancement	no	PML lesion, right frontal lobe
16	pre-IRIS (-14 d.)	6	multilobar, inceasing extension compared to preceeding MRS	frontal lobes in both hemispheres, temporal & occipital right hemisphere	yes	no	yes, punctate& patchy, rim enhancement	no	PML lesion, right frontal lobe
17	pre-IRIS (-10 d.)	6	multilobar, increasing extension	thalamus,cerebral peduncle, right hemisphere	no	no	yes, diffuse & punctate	no	PML lesion, right Thalamus
18	pre-IRIS (-40 d.)	6	multilobar, increasing extension	occipital lobe, right hemisphere	no	no		no	PML lesion right occipital lobe
18	IRIS (0 d.)	6	multilobar, increasing extension compared to preceeding MRS	occipital & parietal & temporal lobe, right hemisphere	no	yes	yes, punctate& patchy, rim enhancement	no	PML lesion right occipital lobe
19	pre-IRIS (-30 d.)	4	unilobar increasing extension	cerebellum &middle cerebellar peduncle, left hemisphere	yes	no		no	PML lesion left cerebellar lobe
19	IRIS (0 d.,	4	unilobar increasing	cerebellum & middle cerebellar peduncle, left	yes	yes	yes, punctate&	no	PML lesion left

	beginning of CE)		extension compared to preceeding MRS	hemisphere & brainstem			patchy		cerebellar lobe
19	E- post-PML (4 m.)	6	multilobar, increasing compared to preceeding MRS	cerebellum left hemisph. & brainstem & middle cerebellar peduncles both hemispheres	yes	no		no	PML lesion left cerebellar lobe
19	E- post-PML (8 m.)	6	multilobar, decreasing compared to preceeding MRS	cerebellum left hemisph. & brainstem & middle cerebellar peduncles both hemispheres	yes	no		yes	PML lesion left cerebellar lobe
20	IRIS (0 d.)	4	Multilobar, increasing extension	parietal and occipital lobe, left hemisphere	yes	yes	yes, punctate& patchy, rim enhancement	yes	PML lesion, left parietal lobe

Abbreviations: MRS=MR-spectroscopy, PML=progressive multifocal leukoencephalopathy, CE=contrast enhancement; IRIS= immune reconstitution inflammatory syndrome,

a: PML group (disease duration): pre-IRIS= before onset of IRIS, IRIS=at IRIS with CE, E-post-PML=early post PML (< 19 months), L-post-PML=late post-PML (> 24 months); disease duration= time before / after IRIS in days (d.) or months (m.)

b: PML lesion extension score (maximum 9), assessed on axial FLAIR images at time of MRS: PML lesion affecting 1= single gyrus, 2= two gyri, 3= single lobe, 4= multiple lobes, 5= one hemisphere, 6= multiple lobes in both hemispheres, 7= one hemisphere and one contralateral lobe, 8= one hemisphere and multiple contralateral lobes, 9= both hemispheres

1.1

frontiers

1.2 Supplementary Figures

Supplementary Figure 1. Dependence of $NAA_{PML/NAWM}$ (upper panel) and $Cr_{PML/NAWM}$ (lower panel) on the time difference between the MRS examination and the onset of IRIS. Grey symbols: all patients who had a single MRS examination; red symbols: patient no. 1, green symbols: patient no. 16, blue symbols: patient no. 18, orange symbols: patient no. 19 (here: IRIS=early IRIS with beginning of contrast enhancement), light blue symbols: patient 7. Dotted lines: linear regression lines of single metabolite ratios and time difference before IRIS.

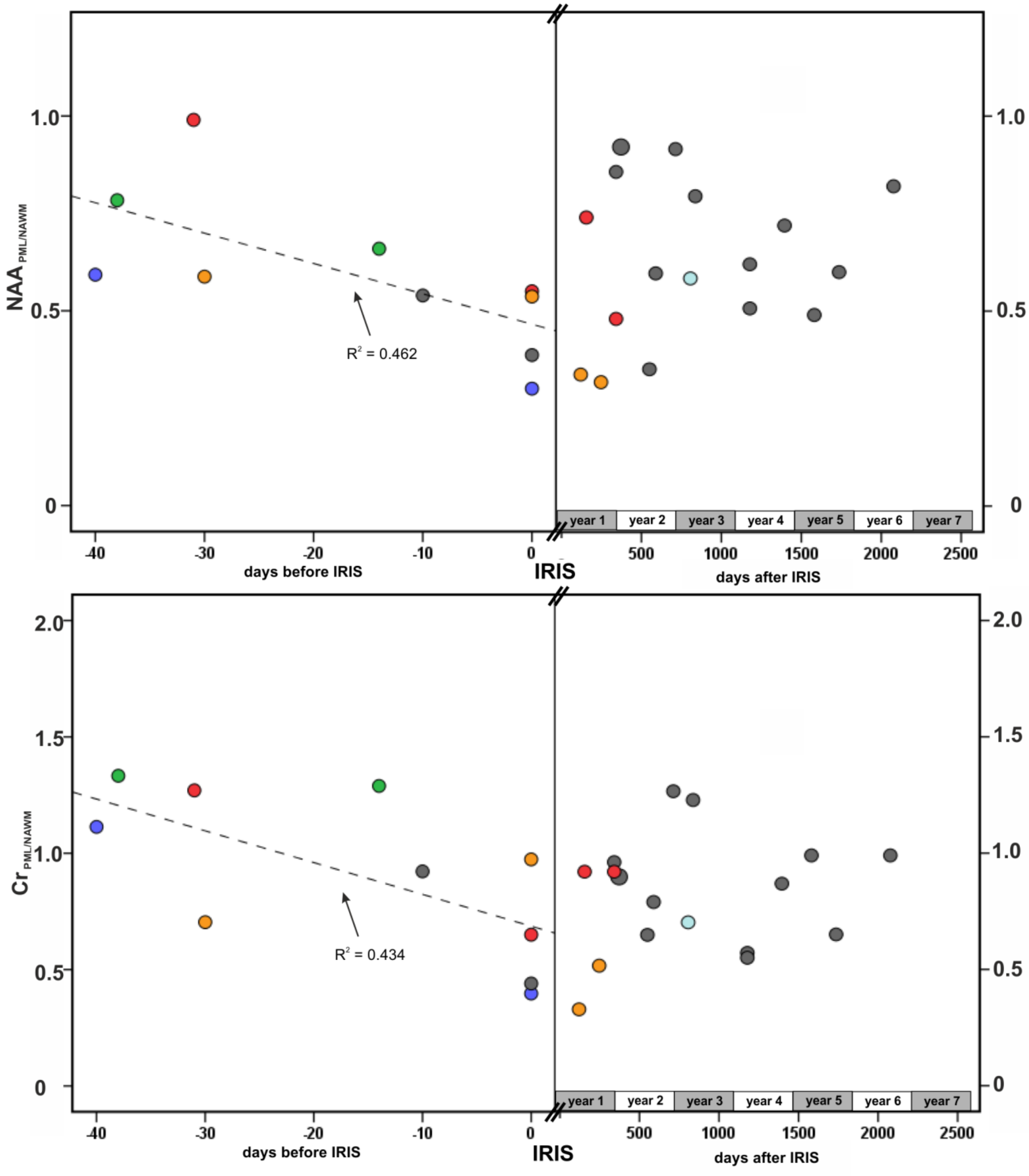

Supplementary Figure 22. Dependence of $Cho_{PML/NAWM}$ (upper panel) and $Lip_{sum\ PML/NAWM}$ (lower panel) on the time difference between the MRS examination and the onset of IRIS. Grey symbols: all patients who had a single MRS examination; red symbols: patient no. 1, green symbols: patient no. 16, blue symbols: patient no. 18, orange symbols: patient no. 19 (here: IRIS=early IRIS with beginning of contrast enhancement), light blue symbols: patient 7. Dotted lines: linear regression lines of single metabolite ratios and time difference before IRIS.

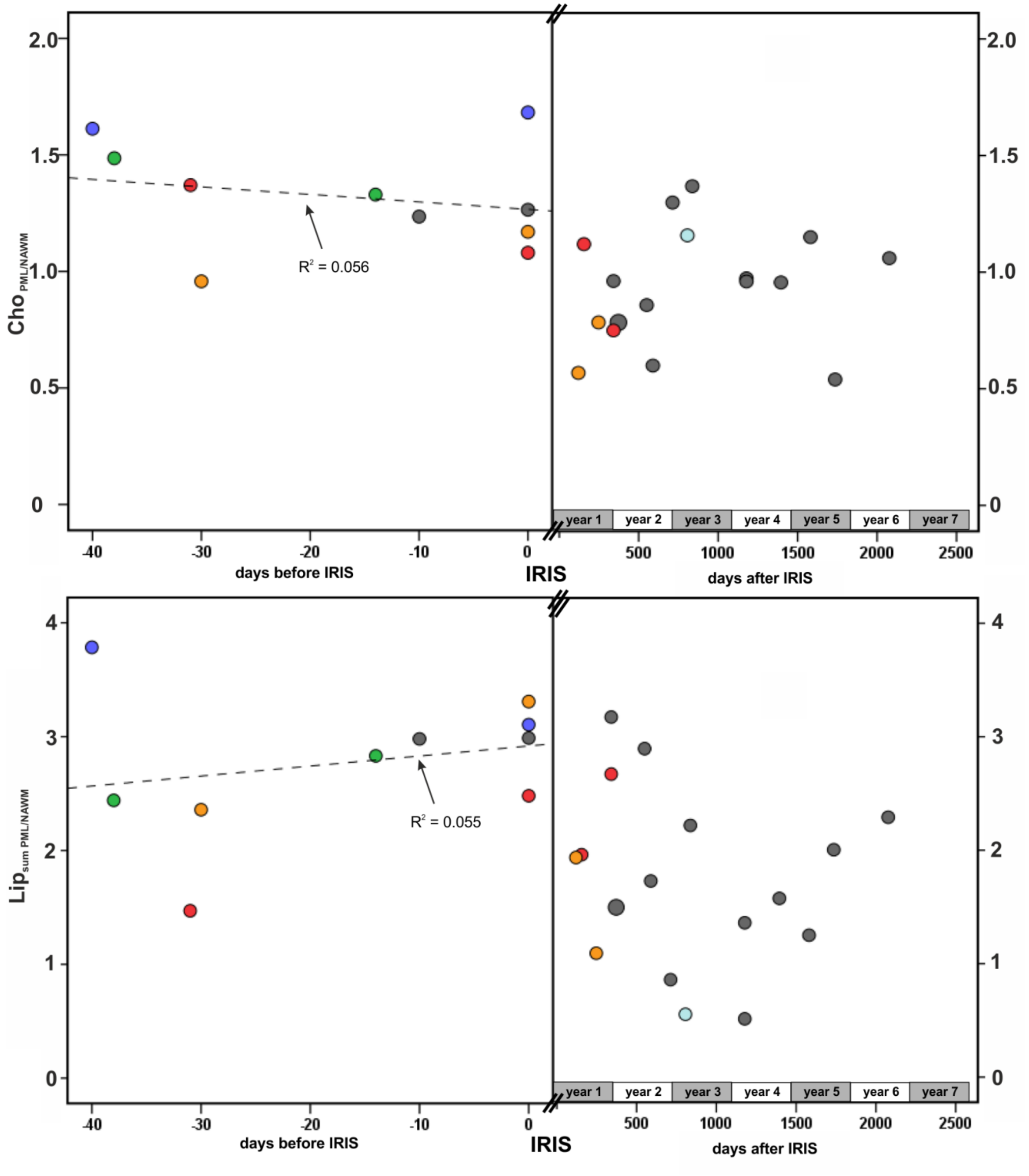

Supplement: Supplementary file 1 [file Presentation_1.PDF]
